# Fetal Growth Is Associated with Amniotic Fluid Antioxidant Capacity, Oxidative Stress, Minerals and Prenatal Supplementation: A Retrospective Study

**DOI:** 10.3390/antiox14020184

**Published:** 2025-02-05

**Authors:** Mozhgan Kohzadi, Stan Kubow, Kristine G. Koski

**Affiliations:** School of Human Nutrition, McGill University, MacDonald Campus, Sainte-Anne-de-Bellevue, QC H9X 3V9, Canada; mozhgan.kohzadi@mail.mcgill.ca (M.K.); stan.kubow@mcgill.ca (S.K.)

**Keywords:** multivitamin-mineral supplements, amniotic fluid, oxidative stress, antioxidant capacity

## Abstract

Background: Associations of antioxidants in prenatal over-the-counter multivitamin-mineral (OTC MVM) supplements with in-utero oxidative stress (OS), antioxidant capacity, and fetal growth are limited. Our objectives were to determine if five fetal ultrasound measurements [biparietal diameter (BPD), head circumference (HC), abdominal circumference (AC), femur length (FL), and estimated fetal weight] were associated with OTC MVM supplements and with minerals, biomarkers of OS, and total antioxidant capacity in amniotic fluid (AF). Methods: For this retrospective study, 176 pregnant women who had undergone age-related amniocentesis for genetic testing were included. Questionnaires recorded prenatal OTC MVM supplementation (yes, no). Ultrasound measurements for early (16–20 weeks) and late (32–36 weeks) gestation were extracted from medical charts. AF concentrations for 15 minerals and trace elements and OS biomarkers in AF [nitric oxide (NO), thiobarbituric acid-reactive substances (TBARS), and ferric-reducing antioxidant power (FRAP)] were measured at 12–20 weeks of gestation. Associations of AF minerals, OS biomarkers, and ultrasound measures were analyzed using multiple linear regressions. Results: Positive associations were observed between AF TBARS and seven AF minerals/elements (calcium, copper, magnesium, nickel, strontium, zinc and iron). At 16–20 weeks, AF copper, nickel, strontium, and selenium were positively associated with BPD, HC, AC, and FL, respectively, NO was positively associated with FL, and FRAP was inversely associated with estimated weight. At 32–36 weeks, calcium was positively associated with BPD and chromium and arsenic were negatively with HC. At 16–20 weeks, higher AF FRAP was inversely associated with FL and this exposure continued to be inversely associated with estimated weight at 32–36 weeks. Conclusions: Concentrations of AF minerals, trace elements and biomarkers of OS and in-utero antioxidant capacity were linked to specific ultrasound measurements at different stages of gestation, suggesting a complex interplay among in utero OS, antioxidant capacity, OTC MVM supplements, and fetal growth.

## 1. Introduction

Oxidative stress (OS), an imbalance between production of reactive oxygen/nitrogen species (ROS and RNS) and antioxidant defense systems, have been implicated in the pathophysiology of miscarriage, pre-eclampsia, intrauterine growth restriction (IUGR), premature rupture of the membranes, and low birth weight [1,2,3]. In this regard, ultrasound measures of intrauterine stress have demonstrated positive relationships of pathological and pre-pathological scores with blood values for thiobarbituric reactive substances (TBARS), an index of lipid peroxidation [4]. However, there have been contradictory findings regarding the role of OS in complicated pregnancies. Decreased antioxidant capacity has been associated with gestational diabetes, premature birth and IUGR [5,6] whereas preeclampsia was associated with higher plasma total antioxidant capacity [7]. In terms of normal pregnancies, the relationship of OS biomarkers with in-utero fetal growth and ultrasound measurements has received limited attention with contrasting findings. Ferguson et al. (2018) reported an inverse association between OS and mid-gestational fetal head circumference and femur length based on maternal urinary 8-isoprostane and plasma 8-hydroxy-2-deoxyguanosine levels [8]. Conversely, Lindstrom et al. (2012) showed that higher urinary levels of 8-isoprostanes in early pregnancy were associated with greater birth length and chest circumference [9]. Although amniotic fluid (AF) is an important tissue to be explored during pregnancy, only a few studies have investigated the relationship of fetal outcomes with AF antioxidant capacity and OS measures [10,11,12]. One study showed that gestational age and estimated fetal weights were positively correlated with the total antioxidant capacity in AF of healthy pregnancies [13].

Presently, the benefits and harms of universal prenatal supplementation are a topic of debate [14,15]. The common expectation is that multivitamin-mineral (MVM) supplementation will support micronutrient pregnancy requirements and optimal maternal and fetal outcomes [16,17]. On the other hand, there is a growing body of evidence that universal supplementation of iron could lead to adverse pregnancy outcomes associated with oxidative damage and depressed antioxidant status [18,19]. Evidence shows that prophylactic iron supplementation in non-anemic pregnant women was related to increased serum levels of the lipid peroxidation marker malondialdehyde (MDA) and higher levels of the pro-inflammatory high-sensitivity C-reactive protein (CRP) [18]. Conversely, the same study showed that iron supplementation in anemic pregnant women was linked with improved hematological status and decreased inflammation without inducing OS [18], suggesting that prenatal iron supplements, if needed, are beneficial but, if not, excess iron is associated with inflammation and OS.

There are several essential minerals in MVM supplements that are cofactors for antioxidant enzymes including selenium, which is a cofactor for glutathione peroxidase [20], as well as copper, manganese and zinc that are cofactors for superoxide dismutase [21]. Vitamins C and E in MVM supplements also serve as antioxidants [22]. We have previously determined novel relationships between AF mineral content and fetal outcomes. This included positive associations between bi-parietal diameter with AF calcium, head circumference with AF copper and nickel, and femur length with AF selenium. Arsenic was negatively associated with estimated fetal weight, which was modified by prenatal supplement use [23].

To our knowledge, no studies have previously evaluated associations of MVM supplementation (yes, no) with in-utero fetal growth or with AF measures of OS and antioxidant capacity which could provide important insight into determinants of early fetal development. Moreover, the study of the effects of high iron MVM supplements on OS, antioxidant capacity, and AF mineral content on fetal growth has not been considered. The objectives of this study were (1) to define whether AF concentrations of minerals, trace elements and the OS and antioxidant biomarkers of nitric oxide (NO), TBARS, and ferric-reducing antioxidant power (FRAP) were associated with five fetal ultrasound measurements at early (16–20 weeks) and late (32–36 weeks) gestation, and (2) to explore if the presence or absence of OTC MVM could modify fetal growth and AF concentrations of minerals, trace elements, biomarkers of OS, and total antioxidant capacity and their associations with individual ultrasound measurements.

## 2. Materials and Methods

### 2.1. Design, Subject Recruitment and Ethics

From 2002 to 2005, healthy pregnant women 34–42 yrs undergoing age-related amniocentesis in Montreal, Canada, were invited to participate in a study investigating associations of amniotic fluid constituents with ultrasound measurements and pregnancy outcomes. Ethics were obtained from McGill University and from St Mary’s Hospital Centre during mothers’ initial visits for age-related amniocentesis. Their consent permitted the use of the discarded amniotic fluid of only healthy mothers without evidence of genetic abnormalities for measurements of AF constituents. Consent from mothers also allowed researchers to review maternal medical charts postpartum in order to obtain fetal ultrasound measurements (biparietal diameter (BPD), head circumference (HC), abdominal circumference (AC), femur length (FL), estimated fetal weight). Following signed consent, self-reported information about OTC MVM supplementation was obtained from questionnaires. Hospital medical records confirmed supplement usage and maternal characteristics (ethnicity, height, pre-pregnancy weight and BMI, age, and parity) and pertinent infant characteristics (infant sex, birth weight and gestational age, and amniocentesis weeks). Only healthy women with singleton pregnancies who provided information on prenatal supplementation were included. Excluded from the criteria were multiple pregnancies, genetic abnormalities, and mothers with incomplete information about supplement use.

For this retrospective study, 176 pregnant women were included. Principle outcomes included amniotic fluid, OS biomarkers (NO and TBARS), and total AF antioxidant capacity (FRAP) analyzed at the time of AF collection in 2004–2006 and 15 AF mineral and trace element concentrations analyzed in 2018 by ICP-MS. Exposures included supplement use (OTC MVM) and timing of supplementation at both early (16–20 weeks) and late (32–36 weeks) gestation (yes/no) using questionnaires and medical record data collected at the time of recruitment. Two subgroups were defined for OTC MVM supplementation: (1) Yes: mothers took OTC MVM supplements at ≤20 weeks, and (2) No: mothers did not take any supplement at ≤20 weeks of pregnancy. Participants reported using one of two OTC MVM Canadian prenatal supplements (Brand A, Brand B) available at the time of study. Nutrient composition of each supplement was confirmed from product labels or product information found on manufacturer websites. With regards to minerals and trace elements, both OTC MVM supplements contained calcium and 60 mg iron, now considered a therapeutic dose, and were the only two minerals included in both prenatal supplements. Other minerals in Brand A included chromium, copper, iodine, magnesium, manganese, molybdenum, and zinc, as well as selenium, which was included in 2004. Information regarding the micronutrient components of two OTC MVMs can be found in Supplementary Table S1.

### 2.2. Biochemical Measures

#### 2.2.1. ICP-MS Analyses of AF Minerals and Trace Elements

Earlier AF bio banked samples were analyzed by ICP-MS for aluminum, arsenic, calcium, chromium, copper, iron, lead, magnesium, nickel, potassium, rubidium, selenium, strontium, and zinc using a Varian 820 ICP-MS (Analytik Jena; Jena, Germany), equipped with a collision reaction interface as previously described [23]. Blanks, standards, replicates, and external quality control samples were digested within the same batch at a ratio of 1 for every 6 samples. Reference samples were used to establish detection limits (μg/L), and recovery performance was assessed using a water sample from Environment Canada Proficiency Testing Program (Sample FPTM 101-7), and two biological reference materials: urine QM-U-Q1306 and serum QM-S-Q1104 samples from the Institute de Santé Publique du Quebec—Centre de Toxicologie. Recovery rates above 90% were recorded for aluminum, strontium, arsenic, copper chromium, nickel, selenium, strontium, lead, and zinc, and above 85% for iron and rubidium. Detection limits were magnesium 2.47, arsenic 2.26, potassium 10.0, calcium 10.0, nickel 1.32, copper 3.15, zinc 1.80, rubidium 0.93, strontium 1.24, lead 0.81, chromium 1.17, arsenic 1.64, and selenium 3.52.

#### 2.2.2. Analysis of AF OS Biomarkers

NO: Total nitrite and nitrate were assayed using 40 µL of sample, an addition of 10 µL of enzyme co-factors, and 10 µL of nitrate reductase to sample duplicates and standards in 96-well Microtest III plates. The plate was left to react at room temperature for three hours for complete conversion of nitrate to nitrite. After the required incubation time, 50 µL of sulfanilamide (Griess reagent 1) was added, followed by an addition of 50 µL of N-(1-Naphthyl)-ethylenediamine (Griess reagent 2) and the color was allowed to develop for 10 min at room temperature. Reagent blanks were prepared for each sample by adding 200 µL of assay buffer instead of the Griess reagents. The absorbance was read at 540 nm using an Automated Microplate Reader (KC4, Bio-Tek Instruments, Inc., Winooski, VT, USA, 2000). A serially diluted standard curve was constructed to quantitate sample nitrate and nitrite concentrations with a detection range of approximately 2.5–35 µM. The conversion of nitrate to nitrite was nearly 100%.

Thiobarbituric acid-reactive substances (TBARS): The TBARS method is considered a global assay for measuring lipid peroxidation and serves as a useful parameter for general oxidative damage. TBARS concentrations were measured using methods adapted from Asakawa and Matsushita (1979) and Wong et al. (1987) and modified by Liu et al. (2007) [24,25,26]. Samples were heated with 2-thiobarbituric acid (TBA) at a low temperature and high pH resulting in the formation of a 1:2 MDA: TBA colored complex, which was quantified spectrophotometrically at 532 nm in a microplate reader (Series 750, Cambridge Technology, Inc., Cambridge, MA, USA). A standard curve was created with which to quantify the TBARS concentrations in the samples. TMOP (1,1,3,3-tetramethoxypropane) in the stock solution is converted to MDA upon heating and binds to TBA forming TBARS. The intensity of the color increases with increasing concentrations of TMOP, thereby forming the standard curve.

Ferric-reducing/antioxidant power assay (FRAP): We measured total antioxidant power using FRAP according to Benzie and Strain (1999) [27]. The FRAP assay assesses “total antioxidant power” using antioxidants as reductants in a redox-linked colorimetric method. Hence, the Fe^3+^-TPTZ (ferric-tripyridyltriazine) complex is reduced at a low pH to the blue ferrous form, Fe^2+^. The procedure involves mixing 300 µL of freshly prepared FRAP reagent containing 300 mM acetate buffer, pH 3.6, 10 mM TPTZ in 40 mM HCI, and 20 mM FeCh·6H_2_O in the ratio of 10:1:1 with 30 µL of water and 10 µL of sample. The absorbance at 593 nm was taken at 6 and 12 min after incubation at room temperature using an Automated Microplate Reader (KC4, Bio-Tek Instruments, Inc., Winooski, VT, USA 2000). The change in absorbance was calculated and related to the change in absorbance of the standard solution. Aqueous ascorbic acid solutions in the concentration range of 100 to 1000 µM were used for calibration of the FRAP assay. The change in absorbance was linearly proportional to the concentration of antioxidant. FRAP values derived from duplicate analysis were expressed in µM units.

#### 2.2.3. Statistical Analyses

All statistical analyses were performed using STATA 18 (StataCorp. 2023. Stata Statistical Software: Release 18. College Station, TX, USA: StataCorp LLC). To determine demographic characteristics, descriptive statistics were used, and, to identify significant differences in ultrasound measurements, AF mineral/trace elements and OS/antioxidant biomarkers in OTC MVM categories (yes/no) and Student’s *t*-test was used. Multiple regression models in separate models with each ultrasound measurement as the dependent variable and individual minerals and trace elements as independent variables were used to determine the significance of their associations with each dependent variable while controlling maternal age, BMI, and ethnicity that were previously identified as confounders [23,28]. We tested each model once with and once without adjusting for prenatal supplement usage. By comparing models with and without adjustments for prenatal supplement use, we were able to test the effect of OTC MVM prenatal supplement (yes, no) as an independent variable, while controlling gestational age, maternal age, BMI, parity, ethnicity, and infant sex. Significance was established at *p* ≤ 0.05 and for all regression models.

## 3. Results

### 3.1. Maternal and Fetal Characteristics

Subject characteristics are summarized in Table 1. Mothers of an older age (37.1 ± 3.0 years) had an average BMI of 24.5 ± 5.3 kg/m^2^. The majority were primiparous (60%), followed by multiparous women with one (14%), two (14%), or three or more children (12%). Most identified as Caucasian (60%), with smaller proportions of Asian (14%), Black (14%), and other ethnicities (12%). Among infants, 45.7% were male and 54.3% were female, with an average gestational age of 39.5 ± 1.4 weeks and birth weight of 3480 ± 499 g. OTC MVM supplementation patterns differed during early (16–20 weeks) and late (32–38 weeks) gestation. Self-reported information showed that 81% consumed an OTC MVM supplement, whereas 19% did not use any supplement prior to 20 weeks of gestation.

Comparisons of individual fetal ultrasound measurements, AF minerals and OS biomarkers between OTC MVM supplemented and non-supplemented pregnant women at ≤20 weeks of gestation are summarized in Table 2. OTC MVM supplementation resulted in higher biparietal diameter (*p* = 0.044) and head circumference percentiles (*p* = 0.049) and estimated fetal weight (*p* = 0.030) at 16–20 weeks of gestation. AF mineral concentrations did not differ except for zinc (*p* = 0.05), chromium (*p* = 0.006) and iron (*p =* 0.030), which were lower in the AF of mothers consuming OTC MVM supplements in early gestation (Table 2). Differences in AF concentrations of three OS/antioxidant biomarkers revealed no differences for FRAP and TBARS, but there were marginally higher concentrations of NO at ≤20 weeks of gestation in OTC MVM supplemented women (32.7 ± 15.1 vs. 27.1 ± 12.2 (μM), *p* = 0.050) (Table 2).

### 3.2. Associations of OS, Antioxidant Capacity, and AF Minerals with Ultrasound Measurements at Early and Late Gestation

#### 3.2.1. Early (16–20 Weeks)

Multiple linear regression models describing the associations of mineral and trace element concentrations and OS/antioxidant biomarkers with individual ultrasound measurements are shown in Table 3. In addition to controlling known maternal and fetal characteristics, including maternal age, parity, ethnicity, BMI, gestational age, and infant sex, OTC MVM was included in the regression models as an independent variable. The inclusion of OTC MVM did not emerge as significant in any model. However, the models identified inverse associations between BPD and copper (β = −0.10, *p* = 0.036, Adj. R^2^ = 0.66) and FL and selenium (β = −0.24, *p* = 0.001, Adj. R^2^ = 0.20), a weak association of HC with nickel (β = −0.06, *p* = 0.050, Adj. R^2^ = 0.84) and a significant positive association of AC with strontium (β = 0.08, *p* = 0.040, Adj. R^2^ = 0.76) (Table 3A).

With regards to concentrations of OS biomarkers in AF at 16–20 weeks gestation (Table 3A), NO was positively associated with FL (β = 0.27, *p* = 0.002, Adj. R^2^ = 0.19), whereas FRAP was negatively associated with estimated weight (β = −0.13, *p* = 0.035, Adj. R^2^ = 0.76); all models controlled maternal age, parity, ethnicity, BMI, gestational age, and infant sex. These significant associations for FL and estimated fetal weight were not further modified by the inclusion of MVM supplementation (Table 3A).

#### 3.2.2. Late (32–36 Weeks)

In Table 3B, multiple linear regression models investigated the association of AF mineral concentrations and OS biomarkers from early gestation with individual ultrasound measurements at 32–36 weeks; the presence of OTC MVM supplementation did not modify these associations (Table 3B). There was a positive association between BPD and calcium (β = 0.13, *p* = 0.045, Adj. R^2^ = 0.36) but inverse associations between HC and chromium (β = −0.14, *p* = 0.036, Adj. R^2^ = 0.35) and HC and arsenic (β = −0.14, *p* = 0.040, Adj. R^2^ = 0.35). At 32–36 weeks, higher FRAP was inversely associated with both FL (β = −0.24, *p* = 0.009, Adj. R^2^ = 0.55) and estimated weight (β = −0.19, *p* = 0.027, Adj. R^2^ = 0.62), revealing that exposure to OS at mid-gestation was associated with in-utero fetal growth.

#### 3.2.3. Association of Supplements, Elements with OS, and Antioxidant Biomarkers in AF

In Table 4, we explored the possibility that individual AF minerals and the presence/absence of an OTC MVM might be associated with concentrations of TBARS, FRAP, and NO. In all regression models, maternal BMI, age, parity, ethnicity, infant sex, and gestational age did not enter individual models. However, distinct minerals and trace elements were associated with TBARS and FRAP. In amniotic fluid, TBARS were positively associated with seven nutrients at mid-gestation: calcium (β = 0.46, *p* = 0.000, Adj. R^2^ = 0.20), copper (β = 0.49, *p* = 0.000, Adj. R^2^ = 0.23), iron (β = 0.44, *p* = 0.000, Adj. R^2^ = 0.21), magnesium (β = 0.33, *p* = 0.002, Adj. R^2^ = 0.10), nickel (β = 0.36, *p* = 0.001, Adj. R^2^ = 0.12), strontium (β = 0.35, *p* = 0.001, Adj. R^2^ = 0.11), and zinc (β = 0.44, *p* = 0.000, Adj. R^2^ = 0.19). In contrast, there was a negative association of FRAP with arsenic (β = −0.24, *p* = 0.042, Adj. R^2^ = 0.10). For all these associations, only 10% to 23% of the variability of the OS/antioxidant biomarker was explained. Moreover, the presence of OTC MVM in the models did not further modify associations of the OS or antioxidant biomarkers (Table 4).

## 4. Discussion

The investigation of OS in the prenatal period has become increasingly relevant due to the critical role of ROS in fetal development and the pathophysiology of various prenatal complications [29]. We adopted a novel approach to assess OS/antioxidant associations with the in-utero environment and fetal development using early second trimester AF samples and fetal ultrasound measurements from both early and late gestation. Our findings uncovered several novel relationships. At 16–20 weeks, four AF elements (copper, nickel, selenium and strontium) emerged as associated with BPD, HC, AC and FL, respectively, and NO emerged as positively associated with FL, whereas FRAP was negatively associated with estimated weight. At 32–36 weeks, different AF elements emerged as associated with fetal growth measurements, including calcium, which was positively associated with BPD, and copper and arsenic, which were negatively associated with HC. Moreover, higher AF FRAP was negatively associated with FL and continued to be inversely associated with estimated weight at 32–36 weeks of gestation. Finally, when all models were adjusted for OTC MVM supplement usage (yes, no), no significant associations of supplementation with any of the five ultrasound measurements were observed. Collectively, these findings demonstrate associations of fetal ultrasound measurements with redox balance and highlight the dynamic interplay of second trimester amniotic fluid minerals and trace elements with fetal development during both early and late gestation.

### 4.1. Associations of AF Mineral/Trace Elements with OS and Antioxidants

Our study showed positive associations between AF TBARS, a marker of OS, with magnesium, calcium, nickel, copper, zinc, strontium, and iron. In addition, a negative association was observed between total antioxidant capacity and arsenic in AF.

TBARS and Minerals: Our study is the first study to associate human AF mineral concentrations and specific biomarkers of OS and antioxidant status. The association between TBARS and specific minerals can be explained by the role that these trace minerals play in OS and inflammation. OTC MVM brands in this study had 60 mg elemental iron in their formulation, which is above the recommended upper limit value for iron for pregnant women [30]. Excess iron could lead to oxidative damage, depressed antioxidant function [19], or interrupt the absorption of other minerals, including zinc and chromium [31,32]. The imbalance in these minerals, such as increased copper concentrations, may further exacerbate OS due to the release of copper during inflammatory tissue damage [33].

TBARS are considered a good index of lipid peroxidation in biological samples [34]. No previous study has evaluated the association of TBARS with minerals or trace elements in healthy pregnancies using AF; however, the present work coincides with the positive correlations noted between TBARS and zinc, nickel, and iron in the skeletal muscle tissue of dogfish sharks [35]. Although copper, zinc, and iron are essential trace elements, high tissue accumulation of these redox-active metals is associated with excessive ROS generation, inhibition of the activity of antioxidant enzymes, and increased lipid peroxidation [36]. For instance, Pu et al. showed that pigs receiving high amounts of supplemental copper, iron, and zinc, and had a tissue mineral overload of these elements, leading to excessive liver TBARS concentrations [37]. As copper and iron are the most abundant transition metals, disturbances in the cellular homeostasis of these redox active metals have been particularly related to oxidative damage to cellular components, such as mitochondria, leading to increased disease risk [38]. Also, nickel can directly generate ROS from molecular oxygen and suppress the antioxidant system, resulting in lipid peroxidation [39].

The role that specific minerals could play in OS and inflammation during pregnancy is understudied. OTC MVM brands in this study had 60 mg elemental iron in their formulation, which is above the recommended upper limit for iron for pregnant women [30]. Excess iron alone can lead to oxidative damage, depressed antioxidant function [18,19], or directly impede the absorption of other minerals, including zinc and chromium [31,32].

FRAP and arsenic: The negative association between FRAP and AF arsenic concentrations in our study may be attributed to several factors. Firstly, arsenic is known to induce OS [40], leading to the depletion of antioxidants, and thus, can lower FRAP levels, as antioxidants are used up in response to the OS. Secondly, arsenic exposure can affect the absorption and utilization of essential nutrients involved in antioxidant pathways, potentially lowering FRAP levels [41]. In addition, in our previous work, arsenic was negatively associated with estimated fetal weight, and this relationship was modified by prenatal supplement use [23]. We surmised that supplemental folic acid may alleviate arsenic toxicity through arsenic methylation [42]. In the present study, we found a negative association between HC and AF arsenic; although, the OTC MVM supplementation did not modify this association.

### 4.2. Associations of Ultrasound Measurement with AF OS/Antioxidant Biomarkers

A positive association between AF NO and FL in early gestation, and a negative association between FRAP in late gestation, with both FL and estimated weight in early and late gestation, emerged in our study. These findings highlight the sensitivity of these AF OS markers when associated with fetal outcomes with specific ultrasound measurements.

NO and femur length: In our study, we observed marginally higher AF NO concentrations in supplemented women and a positive association between early gestation FL and AF NO concentration. NO can play a dual role with respect to its influence on the fetoplacental vasodilation as well as nitrative stress-induced pathological conditions of pregnancy [8]. Previous animal studies have shown that the inhibition of nitric oxide synthase during pregnancy causes decreased placental and fetal perfusion and, subsequently, results in impaired fetal growth [8]. On the other hand, NO as a free radical can negatively affect vascular function in the face of oxidative/nitrative stress and so lead to adverse pregnancy outcomes [8,43]. In the context of this study, higher AF NO concentrations in the supplemented women in early pregnancy might suggest a beneficial effect of OTC MVM on NO concentrations and its role in placental development, as suggested by Ferguson et al., 2018 [8].

FRAP, femur length, and estimated weight: A previous study showed that higher levels of FRAP and vitamin A in newborn cord blood were positively associated with birth weight and BMI-for-age z-score [44]. In contrast, we observed negative associations between AF FRAP concentrations from early second trimester with (a) FL measured at 32–36 weeks and (b) estimated fetal weight measured at both 16–20 weeks and 32–36 weeks. Although the present findings appear paradoxical, it is noteworthy that ROS play numerous physiological roles in cell defense, metabolism, growth, and differentiation [45]. In support of this contention, higher urinary levels of 8-isoprostanes in early pregnancy were associated with greater birth length and chest circumference [9]

### 4.3. Associations of AF Minerals and Trace Elements with Fetal Growth During Early Gestation (≤20 Weeks of Gestation)

Copper, nickel, and selenium were inversely associated with BPD, HC, and FL, respectively; strontium was positively associated with AC.

Bi-parietal diameter (BPD) and copper: There are limited data regarding maternal copper intakes or concentrations during human pregnancy. Copper contributes to the formation of structural proteins and is part of the antioxidant defense system [46]. Copper can also play an important role in fetal development, as lower copper concentrations have been observed in human placentae of small-for-gestational-age (SGA) infants [47]. In our study, we observed an association between lower BPD at mid-gestation with higher AF copper concentrations. It has been suggested that elevated copper concentrations may negatively impact fetal growth, as high copper can disrupt the absorption or utilization of other essential nutrients for fetal growth, such as zinc [46]. Our results suggest that BPD in early gestation is vulnerable to higher concentrations of copper that may be associated with metabolism of other nutrients.

Head circumference (HC) and nickel: In our study, nickel was associated with reduced HC at 16–20 weeks gestation and, to the best of our knowledge, no other study has evaluated this fetal outcome previously. However, nickel had been ranked as a predictor of birth weight [48] and a more recent study confirmed a negative association between nickel from maternal urine and birth weight [49]. Also, environmental metals, such as lead, cadmium, and mercury, have been identified as potential risk factors for adverse effects on pregnant women and the developing fetus, including low birth weight, reduced birth length and reduced head and chest circumferences [50,51], but it is possible that nickel may need to be added to this list.

Femur length (FL) and selenium: Our data showed that AF selenium was associated with reduced FL by 16–20 weeks of pregnancy. An older study of toxic selenium exposure in rats had indicated that sub-chronic selenium toxicity would lead to reduced body weight and femoral length [52]. Moreover, higher selenium concentrations in placenta have also been associated with intrauterine growth restriction and preterm birth [47]. Selenium is a cofactor for enzymic antioxidant system, and it is possible that, in the context of this study, higher selenium levels may be a compensatory mechanism to overcome OS [53]. In our study, we also found a negative association between higher FRAP at mid gestation and lower FL at 32–36 weeks of gestation, but further studies are required.

Abdominal circumference (AC) and strontium: Findings from a recent prospective cohort study on trimester-specific urinary strontium concentrations during pregnancy that longitudinally assessed fetal growth showed that urinary strontium concentrations in the second trimester of pregnancy were positively associated with HC and AC [54], which agrees with the results of our study showing a positive association between AC and AF strontium. The association between strontium concentrations and fetal growth may be attributed to its capacity to cross the placental barrier [55] and its function in eliminating lipid peroxidation products and preventing oxidative damage [56,57].

### 4.4. Late Gestation (>20 Weeks of Gestation)

In late gestation, different associations between early second trimester AF mineral/trace element concentrations and ultrasound measurements measured later (32–36 weeks) in gestation emerged. These included positive associations between BPD and calcium, and negative associations between HC and both chromium and arsenic. These observations show that distinct AF minerals and trace elements are associated with fetal growth during early and late gestation and underscore the complex interplay between these elements and fetal growth.

Bi-parietal diameter (BPD) and calcium: Maternal consumption of dairy products and individual supplementation with calcium have previously been linked to enhanced bone quality and growth [58,59,60,61]. At the time of our study, the RDA for calcium was 1000 mg and both supplements contained less than 25% of the RDA but AF calcium concentrations were associated with increased BPD at 32–36 weeks, which highlights its biological role in fetal development. A previous study on calcium supplementation at 1.5 g and BPD did not observe a relationship [62], suggesting that the impact of calcium might be synergistic, and that the presence of other vitamins, such as vitamin D, in both of our supplements may be required for maximal effect. Particularly noteworthy in our study was the observation that AF calcium concentrations at 12–20 weeks gestation were linked to higher BPD measurements later in pregnancy, suggesting that regular maternal intake of supplemental calcium throughout pregnancy, which is accompanied by increased fetal swallowing of AF with larger fetuses, may have contributed to enhanced fetal growth during the latter half of pregnancy [63].

Head circumference (HC) and chromium and arsenic: In our study, we found negative associations between AF chromium and arsenic with fetal HC, which have not been explored in previous studies. Chromium-related embryotoxic and fetotoxic effects have been observed in animal studies, including decreased fetal weight, skeletal defects, malformations, and death [64,65,66,67,68]. Others have reported that maternal exposure to chromium resulted in higher maternal urinary chromium with an increased risk of low birth weight in China [67]. Moreover, our previous study showed a negative association between estimated fetal weight and AF arsenic [23], which could be related to OS [41], as an association between reduced AF FRAP with arsenic concentrations was observed.

### 4.5. Consequences of OTC MVM Supplementation on In-Utero Fetal Growth

Currently, there is little evidence that universal prenatal supplementation might be beneficial or problematic in developed countries where supplementation has become increasingly popular [16,69]. The majority of the pregnant women in our study reported OTC MVM use, particularly in early gestation (>80%), which is consistent with trends reported in other studies from Canada [70,71]. Nutritional deficiencies are not expected to be a major concern in developed countries [72]. However, a large cohort study of pregnant women in Quebec, where the study took place, revealed a high prevalence of inadequate dietary mineral intake for iron, magnesium, and zinc, in addition to vitamins D, B6, and folate [71]. Furthermore, in our supplemented group, we observed improved BPD and HC percentiles, as well as higher estimated fetal weight. Both OTC MVMs in our study had over 10 different vitamins in their formulation that could potentially meet a maternal mother’s nutritional requirements if required and support fetal development [72], but further research is warranted to determine if supplementation would increase total antioxidant capacity and improve fetal growth.

The only minerals routinely supplemented in OTC MVMs at the time of the study were iron and calcium, where calcium was provided at only 25% of the required amount but was associated with higher BPD. In contrast, at the time of data collection, popular OTC MVM brands had 60 mg iron in their formulation, which is considered to exceed the reported upper limit of 45 mg indicated by Health Canada [30]. Concerns have been raised regarding excess iron and its adverse impact on maternal and fetal outcomes in iron-replete pregnant women [14,15]. Currently, Health Canada recommends that pregnant women take a multivitamin supplement with 16–20 mg of iron and 400 mcg of folic acid [73]. Canada has lowered its iron content in OTC MVM supplements from 60 mg to 27 mg [70], which is unique among countries worldwide, where many still recommend 60 mg [16]. Excess iron could lead to oxidative damage or interrupt the absorption of other minerals, including zinc and chromium [31,32]. In our study, we found that higher AF iron concentrations were associated with higher AF TBARS, supporting the concerns about iron-induced oxidative damage in-utero.

### 4.6. Strengths and Limitations

Collectively, our results provide evidence highlighting novel associations of AF minerals, trace elements, and OS/antioxidant biomarker concentrations, and their associations with in-utero fetal ultrasound measurements at early and late gestation in healthy pregnant women. In addition, we showed that OTC MVM supplementation can modify some, but not all, AF mineral concentrations and OS/antioxidant biomarkers. Despite these strengths, some limitations deserve comment. Maternal medical charts did not routinely record details regarding maternal supplementation brand/type, pattern of usage, dose, and duration of the supplementation. We did not have the information regarding amniotic fluid vitamin concentrations due to length of AF storage; neither maternal iron status or anemia were recorded in medical charts and dietary intake information was not collected at the time of recruitment. As this is an observational study, we could not identify the pathophysiological pathways involving OS/antioxidants and fetal growth. The present findings, however, provide the groundwork for future studies examining these relationships.

## 5. Conclusions

In conclusion, this study highlights the intricate associations between OS/antioxidant biomarkers, nutritive, and non-nutritive minerals, and trace elements in the context of fetal development. By analyzing the sensitivity and specificity of the above parameters alongside fetal ultrasound measurements, this research provides valuable insights into the biochemical and physiological interactions influencing prenatal growth. Importantly, the present research identified fetal ultrasound measurements as being sensitive and specific to associations among antioxidants, OS biomarkers together with prenatal supplementation. The findings demonstrate that fetal ultrasound measurements could serve as a promising non-invasive biomarker in future research, enabling a deeper understanding of how oxidative balance and mineral status impact fetal health. These results have the potential to inform strategies for improving prenatal care and monitoring.

## Figures and Tables

**Table 1:** Maternal, infant and supplementation characteristics.

Characteristics	Mean ± SD or %
Maternal	
Age, years	37.1 ± 3.01
Pre-Pregnancy weight, kg	64.6 ± 14.1
BMI, kg/m^2^	24.5 ± 5.3
Parity	
0	25%
1	40%
2	25%
≥3	10%
Ethnicity	
Caucasian	60%
Asian	14%
Black	14%
Others ^1^	12%
Amniocentesis, week	15.7 ± 1.1
Infant	
Male	45.7%
Female	54.3%
Gestational age, weeks	39.5 ± 1.4
Birth Weight, g	3480.6 ± 499.2
Supplementation (≤20 weeks)	
OTC MVM ^2,3^	
Yes (*n* = 143)	81%
No (*n* = 33)	19%

^1^: Others (Hispanic, Middle Eastern). ^2^: Over-The -Counter Multivitamin-mineral (OTC MVM, *n* = 176) supplementation information. ^3^: Brand A (Vitamins; Vitamin A = 1500 IU, B-Carotene = 1500 IU, Biotin = 30 mg, Vitamin C = 100 mg, Cobalamin = 12 μg, Vitamin D = 250 IU, Vitamin E = 30 IU, Folic acid = 1 mg, Niacin = 20 mg, Pantothenic acid = 10 mg, Pyridoxine = 10 mg, Thiamin = 3 mg, Riboflavin = 3.4 mg, Minerals; Calcium = 250 mg, Chromium = 25 μg, Copper = 2 mg, Iodine = 0.15 mg, Iron = 60 mg, Magnesium = 50 mg, Manganese = 5 mg, Molybdenum = 25 μg, Zinc = 25 mg) (Brand A has changed its formulation after 2004 and added 25 μg of Selenium to its formulation and decreased iron to 27 mg). Brand B (Vitamins; Vitamin A = 2000 IU, B-Carotene = 4000 IU, Vitamin C = 100 mg, Cobalamin = 5 μg, Vitamin D = 400 IU, Folic acid = 1 mg, Niacin = 20 mg, Pyridoxine = 3 mg, Thiamin = 5 mg, Riboflavin = 3 mg, Minerals; Calcium = 160 mg, Iron = 60 mg).

**Table 2:** Comparison of fetal ultrasound, amniotic fluid mineral/trace element, and OS/antioxidant measurements between supplemented and non-supplemented pregnant women at ≤20 weeks of gestation.

Measurements		OTC MVM ^1^		
	Mean	Yes	No	*p*-Value
Fetal Ultrasound				
Bi-parietal diameter (BPD%)	0.53 ± 0.09	0.53 ± 0.13	0.50 ± 0.12	0.044 *
Head circumference (HC%)	0.46 ± 0.10	0.47 ± 0.10	0.44 ± 0.10	0.049 *
Abdominal circumference (AC%)	0.48 ± 0.12	0.47 ± 0.12	0.49 ± 0.13	ns
Femur length (FL%)	0.55 ± 0.10	0.55 ± 0.10	0.56 ± 0.11	ns
Estimated weight (g)	326 ± 59	330 ± 57	309 ± 65	0.030 *
Amniotic Fluid Minerals (μg/L)				
Aluminum (Al)	18.0 ± 15.4	17.8 ± 16.1	18.9 ± 12.9	ns
Arsenic (As)	4.0 ± 1.6	3.9 ± 1.5	4.2 ± 1.8	ns
Calcium (Ca)	52,293 ± 15,101	52,533 ± 16,095	51,255 ± 9806	ns
Chromium (Cr)	3.2 ± 1.4	3.1 ± 0.9	3.7 ± 2.5	0.006 *
Copper (Cu)	102 ± 39	100 ± 37	108 ± 45	ns
Iron (Fe)	536 ± 302	515 ± 197	625 ± 562	0.030 *
Lead (Pb)	1.6 ± 0.9	1.7 ± 1.1	1.5 ± 0.7	ns
Magnesium (Mg)	13,336 ± 2642	13,457 ± 2756	12,811 ± 2027	ns
Nickle (Ni)	0.7 ± 2.1	0.7 ± 2.2	0.8 ± 1.5	ns
Potassium (K)	142,191 ± 23,079	142,888 ± 24,962	139,168 ± 11,673	ns
Rubidium (Rb)	140 ± 30	141 ± 31	133 ± 22	ns
Selenium (Se)	14.0 ± 5.2	13.9 ± 5.4	14.4 ± 4.1	ns
Silver (Ag)	0.2 ± 0.5	0.2 ± 0.5	0.1 ± 0.4	ns
Strontium (Sr)	17.4 ± 6.9	17.5 ± 7.1	17.1 ± 6.6	ns
Zinc (Zn)	88.9 ± 38.8	86.7 ± 34.6	98.7 ± 53.1	0.050 *
AF OS (μM)				
NO ^2^	31.7 ± 14.7	32.7 ± 15.1	27.1 ± 12.2	0.050 *
TBARS ^3^	3.8 ± 1.5	3.8 ± 1.5	3.7 ± 1.2	ns
FRAP ^4^	860 ± 148	857 ± 141	871 ± 178	ns

^1^: Two-sample *t*-test, supplemented women, (yes, *n* = 143), non-supplemented women, (no, *n* = 33). *: Significant *p*-value ≤ 0.05. ns: not significant. ^2^: *n* = 123 (supplemented, *n* = 100/not-supplemented, *n* = 23). ^3^: *n* = 100 (supplemented, *n* = 72/not-supplemented, *n* = 18). ^4^: *n* = 75 (supplemented, *n* = 59/not-supplemented, *n* = 16).

**Table 3:** Associations of individual ultrasound measurements with amniotic fluid mineral/trace element concentrations and OS/antioxidant biomarkers.

**(A) Ultrasound at 16–20 Weeks ^1^**			
**Elements**	**β**	***p*-Value**	**Adj R^2^**
BPD, mm			
Cu	−0.10	0.036 *	0.66
MVM (yes/no)	0.02	ns	
HC, mm			
Ni	−0.06	0.050 *	0.84
MVM (yes/no)	0.03	ns	
AC, mm			
Sr	0.08	0.040 *	0.77
Infant Sex	0.11	0.008 *	
MVM (yes/no)	−0.01	ns	
FL, mm			
Se	−0.24	0.001 *	0.20
Ethnicity	0.16	0.040 *	
MVM (yes/no)	0.02	ns	
FL, mm			
NO	0.27	0.002 *	0.19
Ethnicity	0.19	0.030 *	
MVM (yes/no)	−0.03	ns	
Estimated Weight, g			
FRAP	−0.13	0.035 *	0.76
Infant sex	0.13	0.031 *	
MVM (yes/no)	0.09	ns	
**(B) Ultrasound at 32–36 Weeks**			
**Elements**	**β**	***p*-Value**	**Adj R^2^**
BPD, mm			
Ca	0.13	0.045 *	0.36
BMI, kg/m^2^	0.19	0.004 *	
Infant Sex	0.16	0.018 *	
MVM (yes/no)	−0.03	ns	
HC, mm			
As	−0.14	0.040 *	0.35
MVM (yes/no)	0.03	ns	
HC, mm			
Cr	−0.14	0.036 *	0.35
BMI, kg/m^2^	0.18	0.010 *	
MVM (yes/no)	0.02	ns	
FL, mm			
FRAP	−0.24	0.009 *	0.55
MVM (yes/no)	−0.04	ns	
EstWt, g			
FRAP	−0.19	0.027 *	0.62
BMI, kg/m^2^	0.26	0.001 *	
MVM (yes/no)	0.03	ns	

^1^: In each multiple regression model (A & B), we controlled maternal age, parity, ethnicity, BMI, Infant sex, and reported gestational age at ultrasound measurements. Only the significant results have been presented in the table, except for the gestational ages at ultrasound measurements, which were significant determinants in all the models. *: Significant *p*-value ≤ 0.05. ns: not significant.

**Table 4:** Associations of amniotic fluid OS/antioxidant biomarkers with mineral/trace element concentrations at 12–20 weeks of gestation.

OS/Antioxidant ^1^	β	*p*-Value	Adj R^2^
Elements			
TBARS ^2^			
Ca	0.46	0.000 *	0.20
Cu	0.49	0.000 *	0.23
Fe	0.44	0.000 *	0.21
Mg	0.33	0.002 *	0.10
Ni	0.36	0.001 *	0.12
Sr	0.35	0.001 *	0.11
Zn	0.44	0.000 *	0.19
FRAP ^3^			
As	−0.24	0.042 *	0.10

^1^: In all regression models, we controlled for maternal age, parity, ethnicity, BMI, infant sex, and reported gestational age at ultrasound measurements. None were significant. We entered MVM (yes/no) in all models, which did not emerge as a significant determinant. *: Significant *p*-value ≤ 0.05. ^2^: *n* = 88, *p*-value = 0.032. ^3^: *n* = 73, *p*-value = 0.050.

## Data Availability

The data presented in this study will be available on request from the first author.

## Supplementary Materials

The following supporting information can be downloaded at: https://www.mdpi.com/article/10.3390/antiox14020184/s1, Supplementary Table S1: Vitamin and Mineral Content (Formula) of Brand A and Brand B Prenatal Multivitamins and Recommended Dietary Allowances for Pregnant Women 31–50 y (Health Canada, 2005 [74]).
