# Supplementary material for: Fetal Growth Is Associated with Amniotic Fluid Antioxidant Capacity, Oxidative Stress, Minerals and Prenatal Supplementation: A Retrospective Study
Source: Antioxidants (Basel). 2025 Feb 5;14(2):184. doi: 10.3390/antiox14020184 (PMC11852346; doi:10.3390/antiox14020184)
Supplement: Supplementary file 1 [file antioxidants-14-00184-s001.zip › antioxidants-3401501-supplementary.pdf]

antioxidants MDPI

Research Article

Supplementary Table S1. Vitamin and Mineral Content (Formula) of Brand A and Brand B Prenatal Multivitamins and Recommended Dietary Allowances for Pregnant Women 31-50y (Health Canada, 2005)

	Brand A		Brand B	RDA/AI[1]
	1998-2003	2004-	1998-2006	
**Vitamins**				
Vitamin A	1500 IU	1500 IU	2000 IU	2567 IU
β-Carotene	1500 IU	1500 IU	4000 IU	ND
Biotin	30 mg	30 mg	--	30 ug
Vitamin C	100 mg	100 mg	100 mg	85 mg
Cobalamin	12 ug	12 ug	5 ug	
Vitamin D	250 IU	250 IU	400 IU	600 IU
Vitamin E	30 IU	30 IU	--	15 mg
Folic Acid	1 mg (1000 ug)	1 mg (1000 ug)	1 mg (1000 ug)	600 ug
Niacin	20 mg	20 mg	20 mg	17 mg
Pantothenic Acid	10 mg	10 mg	--	6 mg
Pyridoxine	10 mg	10 mg	3 mg	ND
Thiamin	3 mg	3 mg	5 mg	1.4 mg
Riboflavin	3.4 mg	3.4 mg	3 mg	1.4 mg
**Minerals**				
Calcium	250mg	250mg	160mg	1000mg/d
Chromium	25ug	25ug	--	30ug
Copper	2mg	2mg	--	1mg
Iodine	0.15mg	0.15mg	--	0.22mg
Iron (ferrous fumatate)	60mg	27mg	60mg	27mg
Magnesium	50mg	50mg	--	360mg
Manganese	5mg	5mg	--	2mg
Molybdenum	25ug	25ug	--	50ug
Selenium	--	25ug	--	60ug
Phosphorus	--	--	--	700mg/d
Zinc	25mg	25mg	--	11mg/d
